# Selection and Validation of Reference Genes for Quantitative Real-Time PCR Analysis in Cockroach Parasitoid *Tetrastichus hagenowii* (Ratzeburg)

**DOI:** 10.3390/insects15090668

**Published:** 2024-09-03

**Authors:** Renke Dong, Fengming Cao, Jincong Yu, Yuan Yuan, Jiahui Wang, Zining Li, Chunxue Zhu, Sheng Li, Na Li

**Affiliations:** Guangdong Provincial Key Laboratory of Insect Developmental Biology and Applied Technology, Institute of Insect Science and Technology, School of Life Sciences, South China Normal University, Guangzhou 510631, China; dongrenke@m.scnu.edu.cn (R.D.); 2022023122@m.scnu.edu.cn (F.C.); 2023023070@m.scnu.edu.cn (J.Y.); 20222521038@m.scnu.edu.cn (Y.Y.); wangjiahui@m.scnu.edu.cn (J.W.); 20202521063@m.scnu.edu.cn (Z.L.); 20212521070@m.scnu.edu.cn (C.Z.); lisheng@scnu.edu.cn (S.L.)

**Keywords:** *Tetrastichus hagenowii* (Ratzeburg), reference genes, gene expression analysis, RT-qPCR normalization

## Abstract

**Simple Summary:**

Parasitoid wasps play a crucial role in controlling sanitary pests such as cockroaches, which pose a threat to human health. *Tetrastichus hagenowii* is an effective parasitoid wasp for the biological control of cockroaches, particularly the American cockroach (*Periplaneta americana*). This study aimed to evaluate the stability of potential reference genes in *T. hagenowii*. Seven candidate reference genes were rigorously selected and their reliability was assessed using RT-qPCR. Stability analysis across different developmental stages of *T. hagenowii* was conducted using five algorithmic methods, ultimately identifying *α-tubulin* as the most stable reference gene. Our research not only provides reliable reference genes to enhance studies on gene expression and functional genomics related to *T. hagenowii* but also offers valuable insights for the regulation of parasitoid wasps in cockroach control strategies.

**Abstract:**

Parasitoid wasps play a crucial role in the efficient control of pests, a substantial menace to human health and well-being. *Tetrastichus hagenowii* (Ratzeburg) stands out as the most effective egg parasitoid wasp for controlling American cockroaches, but accurate and stable reference genes for quantitative real-time polymerase chain reaction of *T. hagenowii* genes are still lacking. In this study, we assessed seven candidate nuclear genes, including *α-tubulin* (*α-TUB*), *elongation factor-1-alpha* (*EF-1α*), *β-actin* (*Actin*), *ribosomal protein 49* (*RP49*), *glyceraldehyde-3-phosphate dehydrogenase* (*GAPDH*), *nicotinamide adenine dinucleotide* (*NADH*), and *elongation factor 2* (*EF2*) of *T. hagenowii*. By analyzing expression stability with four algorithms (Delta Ct, geNorm, NormFinder, and BestKeeper), as well as comprehensive ranking with RefFinder, we identified *α-TUB* as the most stable reference gene for the larval, pupal, female adult, and male adult stages. Subsequently, we estimated the transcript levels of vitellogenin (*Vg*) and cuticle protein (*CP*) after normalization with *α-TUB* across various developmental stages. Significantly higher expression levels of *CP* and *Vg* were observed in pupae and female adults, respectively, consistent with previous findings in other insects. This study offers a reliable reference gene for normalizing transcription levels of *T. hagenowii* genes.

## 1. Introduction

Parasitoid wasps have been successfully used against increasing groups of urban cockroaches, a sanitary pest worldwide [1]. Such biological control could avoid potential issues of environmental pollution, weakened control efficacy, and health risks to residents raised by excessive use of insecticides [2]. *Tetrastichus* (*=Aprostocetus*) *hagenowii* (Ratzeburg) (Hymenoptera: Eulophidae), a parasitoid wasp of cockroach oothecae, is the most promising natural enemy to control cockroaches [1,3,4,5,6]. More than 96% of the oothecae (egg masses) of the American cockroach (*Periplaneta americana*) (Blattodea: Blattidae) were parasitized by *T. hagenowii* wasps in indoor experiments [3]. The control effectiveness of parasitoids can be sustained at 90.8–100% for up to five months post-release [7]. Altogether, *T. hagenowii* completes its life cycle in cockroach oothecae in an efficient way, highlighting its substantial value in cockroach control. Gene expression analysis of *T. hagenowii* is of paramount importance in the exploration of gene function and assists in understanding the interaction mechanism between parasitoid wasps and cockroach oothecae.

Quantitative real-time polymerase chain reaction (RT-qPCR/qPCR), with its capacity to detect and measure minute amounts of nucleic acid in a wide range of samples from numerous sources, is a common approach for gene expression analysis [8,9]. For accurate gene quantification, normalization of RT-qPCR data is absolutely essential. The most common way to perform normalization is to relate the mRNA level of targeted genes to that of a stable reference gene [10]. Housekeeping genes (HKGs), such as *β-actin* (*Actin*), *α-tubulin* (*α-TUB*), and *glyceraldehyde-3-phosphate dehydrogenase* (*GAPDH*), are commonly expressed at relative stable levels and are thus widely used as reference genes in insects [8,11,12]. However, it should be noted that the stability of reference genes may not always remain constant under different conditions. In the fruit fly *Drosophila melanogaster*, *rpL18* (*ribosomal protein L18*) exhibits the most stable expression in flies exposed to methanol, whereas treated with ethyl acetate, *rpL18* shows the least stable expression among the tested genes [13]. In the fall armyworm *Spodoptera frugiperda*, *β-1-tubulin* exhibits stability in larval tissues but instability across developmental stages, under varied temperature or starvation stress [14]. In the parasitoid wasp *Cotesia chilonis*, *ribosomal protein 17* and *10* are the optimal reference genes in lower temperature treatment, whereas *18S rRNA*, *histone 3*, and *Actin* are more appropriate in higher temperature experiments [15]. Therefore, it is essential to identify desirable reference genes that maintain a stable expression for functional gene expression analysis under different experimental conditions.

Currently, there is still a lack of studies evaluating reference genes for parasitoid wasps including *T. hagenowii*. To fill the knowledge gap, we evaluated seven commonly used nuclear HKGs (*α-TUB*, *EF-1α*, *Actin*, *RP49*, *GAPDH*, *NADH*, and *EF2*) for their suitability as reference genes in different developmental stages of *T. hagenowii*. We employed five statistical algorithms, including Delta Ct, geNorm, NormFinder, BestKeeper, and a ranking RefFinder, to determine their expression stability. This study provides a reliable reference gene for parasitoids of cockroaches to quantify functional gene expression.

## 2. Materials and Methods

### 2.1. Insect Rearing and Sample Preparation

*Tetrastichus hagenowii* was originally separated from the colony of laboratory cockroach oothecae and maintained for approximately 45 successive generations for nearly 5 years in the Institute of Insect Science and Technology (Guangzhou, China). Fresh oothecae (0–3 days), produced by female cockroaches, were prepared for parasitism. The female and male adult wasps were fed a diet of 10% honey water in a Petri dish with a diameter of 20 cm at 27 ± 1 °C with a relative humidity of 75 ± 10% and 14 h of light daily. The larvae and pupae of *T. hagenowii* were carefully removed from the cockroach oothecae, and the male and female adults were collected within two hours after emerging from the oothecae. These samples were flash frozen in liquid nitrogen and stored at −80 °C until subsequent experiments.

### 2.2. Candidate Reference Genes and Primer Design

A total of seven candidate reference genes (*α-TUB*, *EF-1α*, *Actin*, *RP49*, *GAPDH*, *NADH*, and *EF2*) were selected, which are commonly used for RT-qPCR analyses in other insect species [16]. The nucleotide sequences of these genes in *D. melanogaster* (GenBank IDs: *α-TUB*, NM_079540.6; *EF-1α*, NM_058027.5; *Actin*, NM_078497.4; *RP49*, NM_170460.2; *GAPDH*, NM_057219.4; *NADH*, NM_001275892.1; *EF2*, NM_165395.2) and the corresponding homologs in *Nasonia vitripennis* (jewel wasp) were aligned using BioEdit 7.0 [17]. Based on conserved sequences, gene-specific primers were designed using the NCBI Primer-Blast online tool (https://www.ncbi.nlm.nih.gov/tools/primer-blast/, accessed on 15 August 2021). The optimal amplification of 80–250 bp fragments with a clear single specific band was chosen to test the stability of reference genes.

### 2.3. RNA Isolation and cDNA Synthesis

Approximately 40 larvae, 40 pupae, 30 female adults, and 30 male adults were pooled to constitute one biological replicate; four biological replicates were prepared for each developmental stage. Total RNA was isolated from the whole bodies of the collected samples following the standard Trizol reagent protocol (Takara, Tokyo, Japan). The concentration and quality of total RNA were determined using a NanoDrop ONE spectrophotometer (Thermo Scientific, Waltham, MA, USA) and via gel electrophoresis, which was used to assess RNA integrity. RNA was treated with DNase I (Qiagen, Valencia, CA, USA) to remove genomic DNA contamination before cDNA synthesis. Two micrograms of total RNA were reversely transcribed to generate cDNA using M-MLV reverse transcriptase and Oligo (dT) primers (Promega, Madison, WI, USA) following the manufacturer’s instructions.

### 2.4. Absolute Quantitative Real-Time PCR

To evaluate the amplification efficiency of the designed primers, absolute RT-qPCR was applied on the basis of standard curves. We used equal amounts of cDNA from equally pooled cDNA samples from the larval, pupal, female adult, and male adult stages as templates for PCR cloning. Each reaction was carried out in a final volume of 50 μL, including 2 μL of cDNA, 25 μL of 2× Hieff^®^ PCR Master Mix (With Dye) (Yeasen, Shanghai, China), and 1 μL of each primer pair (10 μM). The thermocycling program consisted of an initial denaturation at 95 °C for 4 min, followed by 35 cycles of 95 °C for 10 s, 56 °C for 20 s, and 72 °C for 30 s. PCR products of the reference genes were purified using the SteadyPure PCR and Gel DNA Purification Mini Kit (Accurate Biology, Changsha, China). The purified DNA fragments were subsequently ligated into a pMD18-T vector (Takara, Tokyo, Japan) using Solution I. For the ligation reaction, a mixture containing 0.5 μL of the pMD18-T vector, 2 μL (1000 ng) of insert DNA, and 5 μL of Solution I was prepared, with nuclease-free water added to achieve a final volume of 10 μL. This mixture was incubated overnight at 16 °C. Following ligation, the recombinant plasmids were transformed into *Escherichia coli* DH5α strain via a standard heat-shock method. Specifically, the competent cells were combined with the ligation mixture and incubated on ice for 30 min. This was followed by a heat shock at 42 °C for 90 s and a subsequent incubation on ice for 5 min. The cells were then allowed to recover in SOC medium at 37 °C for 1 h before being plated on LB agar containing the appropriate antibiotic for selection. Recombinant plasmids were quantified using a NanoDrop ONE spectrophotometer (Thermo Scientific, Waltham, MA, USA). A series of ten-fold dilutions from 1 to 10^−7^ ng μL^−1^ of the recombinant plasmid for each gene was prepared. The amount of cDNA of the reference gene in each diluted sample was calculated as copies per microliter using the following equation [18]:

Copies/μL = (6.02 × 10^23^) × (ng/μL × 10^−9^)/(DNA length × 660) (1)

Ct values (Y axis) were plotted against the logarithm of the number of copies (X axis) for each diluted sample to generate the standard curve for each gene. The regression coefficient (*R*^2^) derived from the linear regression equation was used to evaluate the standard curves. The PCR amplification efficiency (*E*) was calculated by the slope of the standard curve according to the following equation [11]:

*E* = 10^−(1/slope)^ − 1 (2)

### 2.5. RT-qPCR Procedure

RT-qPCR was used to measure expression profiling of each candidate gene in the cDNA samples on an Applied Biosystem™ QuantStudio™ 6 Flex Real-Time PCR System (Thermo Scientific, Waltham, MA, USA), as described previously [19]. In short, each reaction was carried out in a final volume of 20 μL containing Hieff^®^ qPCR SYBR Green Master Mix (Low Rox Plus) (Yeasen, Shanghai, China), 2 μL of cDNA template, and 1 μL of each primer pair (10 μM). The two-step thermocycling program was 98 °C for 3 min, followed by 40 cycles of 98 °C for 15 s and 58 °C for 20 s. The primers for each gene are listed in Table 1. The Ct value for each gene was obtained and measured as the mean ± standard error (SE). The RT-qPCRs were performed in four biological replicates each with three technical replicates. The RT-qPCR products for each gene were subjected to 1% agarose gel electrophoresis for validation, with a voltage setting of 120 V and a duration of 25 min.

### 2.6. Stability Analysis

In the context of RT-qPCR, the stability of a reference gene is defined by the consistency and reliability of its expression levels across various experimental conditions, tissues, and developmental stages. To evaluate the stability of the seven reference genes, the average of the raw Ct values was analyzed using the comparative Delta Ct, BestKeeper, NormFinder, and geNorm algorithms and finally ranked comprehensively using the RefFinder algorithm. These tools are based on different statistical algorithms and may produce different rankings in stability within the same reference gene study. For the Delta Ct method, gene stability is ranked based on the average SD, and the gene with the lowest average SD is considered the most stable [20] Similarly, in the BestKeeper algorithm, the most stable reference gene should have the lowest SD values [21]. In the NormFinder method, the raw Ct value should be transformed to a relative quantitative value (2^−ΔΔCt^) prior to analysis. A variation value named the SV was obtained using NormFinder analysis and the most stable gene has the lowest SV [20]. geNorm analysis uses the converted relative quantitative values, and an expression stability measurement (M) value is calculated, with candidates ranked by M value (within a cut-off threshold of 1.5), a smaller value indicating better stability [11]. The stability values for the four algorithms are independent from each other. RefFinder integrates results from multiple algorithms (including Delta Ct, BestKeeper, NormFinder, and geNorm) to provide a comprehensive assessment of gene stability. RefFinder calculates a geometric mean (GM) of the rankings from these methods, providing a final ranking of the reference genes based on their overall stability [22].

### 2.7. Validation of Candidate Reference Genes across Different Developmental Stages

The nucleotide sequences of the vitellogenin (*Vg*) and cuticle protein (*CP*) genes in *Phymastichus coffea* (coffee berry borer parasitoid) (GenBank IDs: *Vg*, XM_058943345.1 and *CP*, XM_058943064.1) were used to evaluate the stability of the candidate reference genes. The primer sequences for the target genes were as follows: *Vg* forward (5′-CTGAGTACGAAAAATACTCGGT-3′), *Vg* reverse (5′-ATAAGTTTCTGCTTGCGTTC-3′), *CP* forward (5′-CGCCATTAACATCGCATT-3′), and *CP* reverse (5′-TCCTTACAGCCTCGTAGT-3′). The average transcript levels of *Vg* and *CP* across developmental stages were calculated using the 2^−ΔΔCt^ method, based on four biological replicates. Statistical analysis was conducted using one-way ANOVA followed by Tukey’s test for multiple comparisons. Two separate one-way ANOVA analyses were performed, one for *Vg* and one for *Cp*, with developmental stages (larva, pupa, female adult, and male adult) being treated as fixed effects in each analysis.

### 2.8. Statistical Analysis

Four biological replicates were prepared and assayed for each group of experiments. Ct data were exported to Microsoft Excel 2021 to compute the arithmetic mean and SD for each gene. Subsequently, Ct data were exported to GraphPad Prism 8.0 (GraphPadSoftware, San Diego, CA, USA) to calculate medians and to generate box plot and column graphs.

## 3. Results

### 3.1. Specificity and Efficiency of Reference Gene Primers

The primers for RT-qPCR were designed for each candidate reference gene, with the sizes of the resulting amplification products ranging from 93 bp (*GAPDH*) to 249 bp (*Actin*) (Table 1). To evaluate the amplification efficiency of these primers, the seven purified plasmid templates were used to acquire the standard curve with an absolute RT-qPCR assay. The analysis of standard curves resulted in an amplification efficiency (*E*) ranging from 90.60% (*EF-1α*) to 98.79% (*EF2*) (Table 1), while the regression coefficient (*R*^2^) ranged from 0.991 (*GAPDH*) to 0.999 (*RP49*) (Table 1).

**Figure 1:**
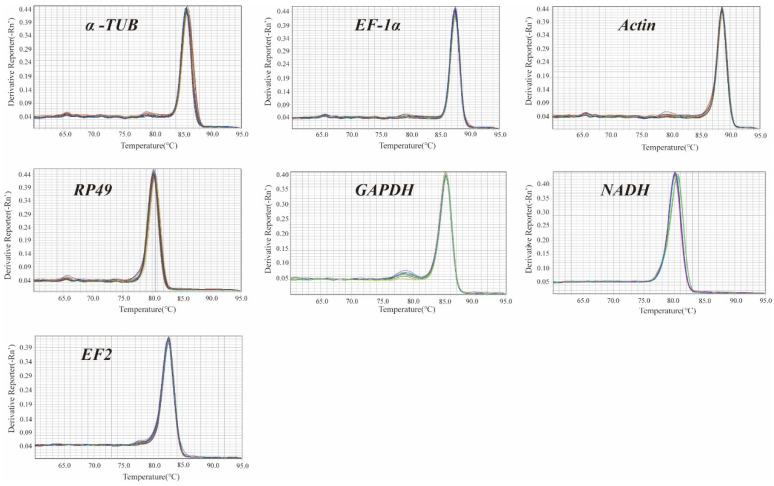
Melting curve to verify the reference gene amplification efficiency and uniformity. A single peak indicates a single RT-qPCR product. *n* = 6 for each developmental stage.

**Figure 2:**
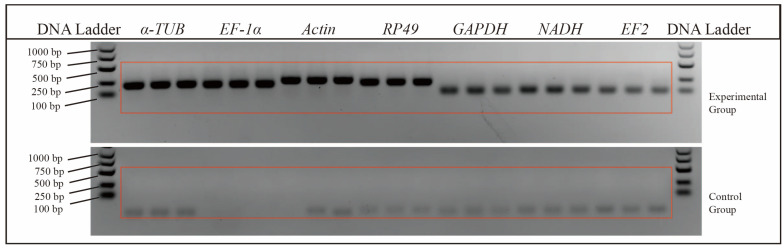
Agarose gel electrophoresis to test the amplification of RT-qPCR primers for candidate reference genes. The RT-qPCR product size for each gene was shown in Table 1. Three biological replicates of mixed samples from the larval, pupal, female adult, and male adult stages were prepared for each gene. The figure shows DNA bands for cDNA samples and the absence of bands for water negative controls.

Melting curve analysis in RT-qPCR revealed a single peak for each reference gene (Figure 1). Furthermore, a single band with the expected size was observed for each primer pair in agarose gel electrophoresis, indicating the presence of single and specific products (Figure 2). These findings suggest successful screening of highly specific and efficient primers [20].

### 3.2. Expression Level of Reference Genes in Larvae, Pupae, and Adults of T. hagenowii

**Figure 3:**
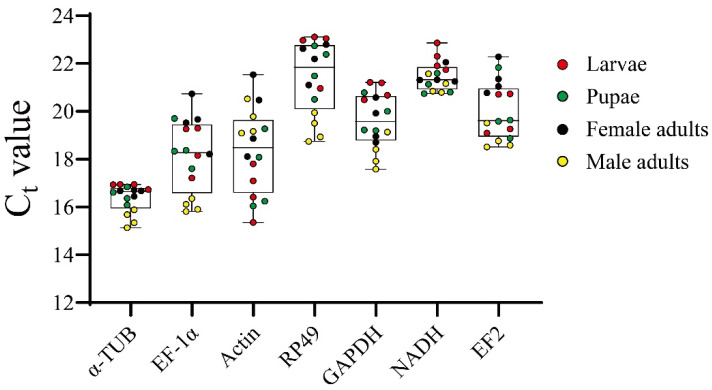
Ct values of the candidate genes evaluated in larvae, pupae, and adults of *T. hagenowii***.** The upper and lower whiskers indicate the maximum and minimum values, respectively. The upper and lower edges of the box represent the upper and lower quartiles, while the middle black line signifies the median. The length of each graphic (whiskers) corresponds to its variation. *n* = 4 for each developmental stage.

The stability of the seven reference genes was investigated across different developmental stages of *T. hagenowii* (Figure 3). The median cycle threshold (Ct) values ranged from 16.645 for *α-TUB* to 21.840 for *RP49*. The *α-TUB* gene demonstrated the highest expression level due to the lowest median Ct value, with a minimum of 16.375 ± 0.582, whereas *NADH* showed the lowest mRNA transcript levels with a maximum Ct value of 21.465 ± 0.601 among the examined reference genes (File S1). Notably, variation in the Ct range is another key criterion for evaluating expression stability. The *α-TUB* gene exhibited the lowest Ct range, at only 1.812 cycles, followed by *NADH* at 2.128 cycles, while the *Actin* gene showed the largest discrepancy, at 6.184 cycles. This indicates that *α-TUB* and *NADH* showed smaller fluctuation ranges than the other five reference genes. The expression profile analysis showed that *α-TUB* and *NADH* were the two most stable genes among the seven candidate reference genes (Figure 3).

### 3.3. Comprehensive Ranking of Candidate Reference Genes across Different Developmental Stages of T. hagenowii

To determine the most reliable reference gene across various developmental stages of *T. hagenowii*, we employed four algorithms—Delta Ct, BestKeeper, NormFinder, and geNorm—collectively within the RefFinder framework.

#### 3.3.1. Delta Ct Method

**Figure 4:**
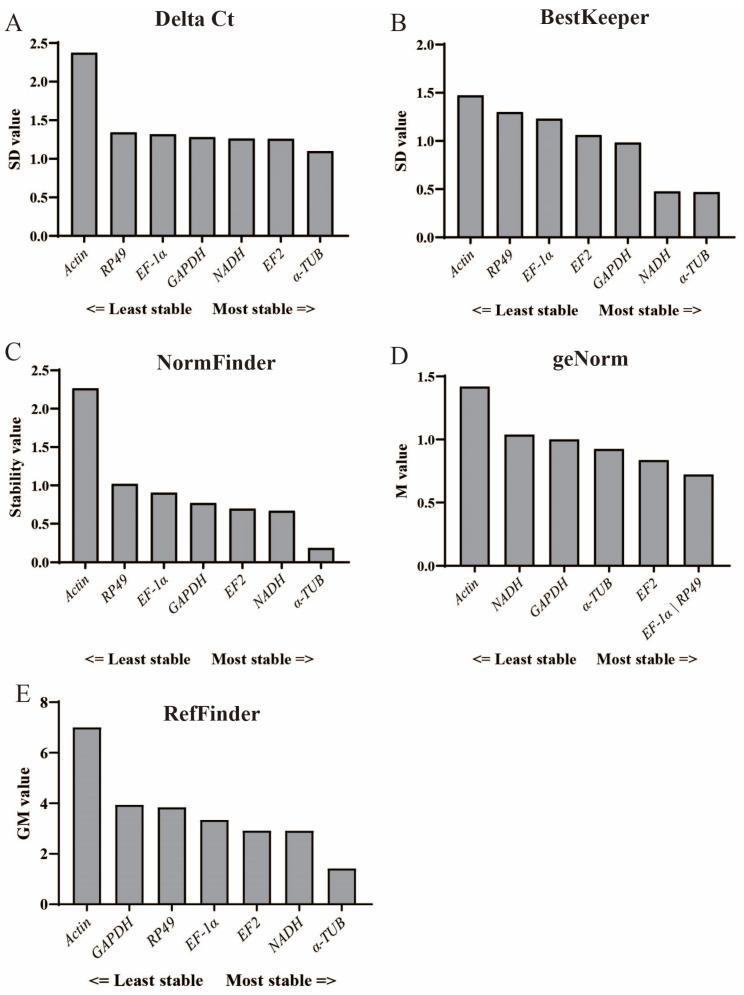
Stability rankings of the seven candidate reference genes in *T. hagenowii* calculated separately by Delta Ct, BestKeeper, NormFinder, geNorm, and RefFinder. The stability values and data are presented from the least stable (left) to the most stable gene (right). (**A**). Delta Ct; (**B**). BestKeeper; (**C**). NormFinder; (**D**). geNorm; (**E**). RefFinder.

The Delta Ct method calculates the average SD (standard deviation) of differences in Ct values of the given genes. Here, the most stably expressed gene was α-*TUB* (average SD is 1.099). In contrast, *Actin* exhibited the least consistency with an SD of 2.373 (Figure 4A). Additionally, the remaining candidate genes were ranked in terms of stability as follows: *EF2* > *NADH* > *GAPDH* > *EF-1α* > *RP49* (Figure 4A).

#### 3.3.2. BestKeeper Method

Utilizing the BestKeeper algorithm, which prioritizes genes with the lowest SD as the most stable, *α-TUB* and *NADH* emerged as the top-ranking reference genes across all samples, with SD values of 0.47 and 0.476, respectively (Figure 4B). In contrast, *GAPDH*, *EF2*, *EF-1α*, and *RP49* exhibited comparatively higher SD values, further highlighting the robust stability of *α-TUB* and *NADH*. Conversely, *Actin* stood out as the least stable reference gene, characterized by a notably higher SD of 1.472.

#### 3.3.3. NormFinder Method

NormFinder analysis yields a variation value termed as the stability value (SV), where the gene with the lowest SV is identified as the most stable. According to the NormFinder results, *α-TUB* exhibited the highest stability (SV = 0.185) (Figure 4C). Furthermore, the remaining genes ranked in the following order: *NADH* > *EF2* > *GAPDH* > *EF-1α* > *RP49* > *Actin* in terms of stability of expression.

#### 3.3.4. geNorm Method

geNorm analysis identified *EF-1α* and *RP49* as the most stable genes, both with a stability value (M) of 0.722. Conversely, *Actin* exhibited the lowest stability with an M value of 1.419 (Figure 4D). Moreover, *EF2* and *α-TUB* displayed superior stability compared to *GAPDH* (1.0 for M value) and *NADH* (1.038 for M value), with stability values of 0.837 and 0.924, respectively.

#### 3.3.5. RefFinder Method

The RefFinder method indicated that *α-TUB*, with a geometric mean (GM) value of 1.414, emerged as the most stable reference gene in all samples (Figure 4E). Following *α-TUB*, *NADH* (2.913 for GM value) and *EF2* (2.914 for GM value) were identified as the second and third most stable genes, respectively. Additionally, *EF-1α* ranked fourth in terms of stability with a GM value of 3.344, while the remaining genes were ordered as *RP49* > *GAPDH* > *Actin*.

### 3.4. Optimal Reference Gene across Different Developmental Stages

In summary, *α-TUB* was identified as the most suitable reference gene across all samples. Specifically, Delta Ct, BestKeeper, and NormFinder analyses demonstrated the highest stability of *α-TUB*, while geNorm analysis identified *α-TUB* as the third most stable gene (Figure 4). Furthermore, RefFinder analysis indicated that *α-TUB* had the lowest GM value, solidifying its status as the most appropriate gene for standardization (Figure 4E).

On the other hand, *Actin*, which exhibited the highest SD value in Delta Ct and BestKeeper analyses, the highest SV value in NormFinder analysis, the highest M value in geNorm analysis, and the highest GM value in the comprehensive RefFinder analysis, emerged as the most unstable gene across all assessments. Therefore, *Actin* is not suitable for use as a reference gene in this study.

### 3.5. Validation of Candidate Reference Genes across Different Developmental Stages

**Figure 5:**
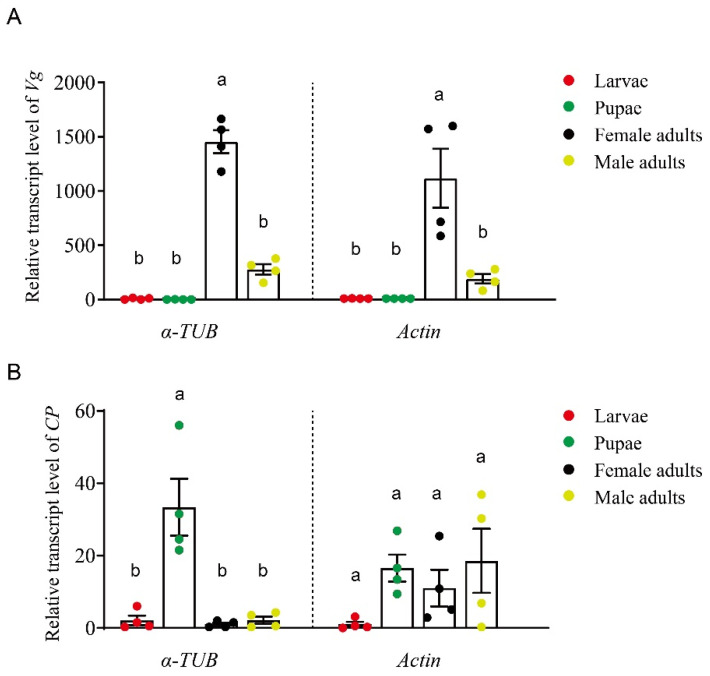
Relative expression levels of *Vg* (**A**) and *CP* (**B**) across developmental stages in *T. hagenowii*. Four biological replicates were prepared for each developmental stage analyzed using RT-qPCR. Transcript levels were normalized with *α-TUB* or *Actin* using the 2^−ΔΔCT^ method and are presented as ratios relative to that of larvae (mean = 1). Columns represent mean values, with error bars indicating standard error (SE). One-way ANOVA was used to assess differences among developmental stages. Different letters above bars mean significant difference between different groups.

The expression patterns of vitellogenin (*Vg*) and cuticle protein (*CP*) genes in various developmental stages of *T. hagenowii* were assessed using the most (*α-TUB*) and least (*Actin*) stable reference genes. A significantly higher transcript level of *Vg* was found in female adults when *α-TUB* was used as the reference gene (Figure 5A) (F = 73.76, *p* < 0.001). Although a similar trend was observed after normalization with *Actin* (F = 8.43, *p* < 0.01), the variability among different replicates in female adults was higher (Figure 5A). With *α-TUB* as the reference gene (F = 10.65, *p* < 0.01), *CP* transcript levels were higher in the pupal stage compared to other developmental stages (Figure 5B). However, no significant differences were observed among the larval, pupal, female adult, and male adult stages when *Actin* was used as the reference gene (Figure 5B) (F = 2.45, *p* = 0.14).

## 4. Discussion

This study evaluated seven candidate reference genes in the parasitoid wasp *T. hagenowii* to establish the optimal normalization strategy for RT-qPCR analysis, laying the groundwork for identifying key genes involved in the parasitization of cockroach oothecae. Notably, we investigated the stable expression of these genes across developmental stages of *T. hagenowii* for the first time. Our research identified *α-TUB* as a stably expressed reference gene, exhibiting the highest expression levels among all seven candidate reference genes (Figure 3 and Figure 4).

*Alpha-TUB* has emerged as a robust reference gene across various insect species, demonstrating remarkable stability throughout different developmental stages and under various physiological conditions. In the fruit fly *Drosophila suzukii* [23] and the oriental armyworm *Mythimna separata* [24], the high stability of *α-TUB* has been highlighted, particularly during various developmental stages. Its consistent expression has been demonstrated during the larval stages of the mosquito *Aedes aegypti* [25], the firefly *Aquatica leii* [26], and the beet armyworm *Spodoptera exigua* [27]. Moreover, it has been identified as a reliable reference gene in adults, such as the moth *Mythimna loreyi* [28] and the kissing bug *Rhodnius prolixus* [29]. In the oriental fruit fly *Bactrocera dorsalis*, *α-TUB* has also been reported to exhibit minimal variation in stability between male and female individuals [30]. These collective findings strongly support the reliability of *α-TUB* as an internal reference gene across diverse insect species and developmental stages, corroborating our experimental observations (Figure 4). In our validation test, the observed higher expression levels of *CP* in the pupal stage (Figure 5B) are consistent with the role of CPs at this stage, which are essential for forming a robust and flexible exoskeleton [31,32,33]. Moreover, the higher *Vg* transcript levels found in female adults (Figure 5A) are reasonable considering that VGs in insects encode the major egg yolk protein precursor in insects [34]. Building upon these findings, *α-TUB* might serve as a crucial reference gene for studying the interaction between parasitic wasps *T. hagenowii* and their host cockroach oothecae.

*Actin*, recognized as the most abundant cellular skeleton protein [35], is frequently utilized as a constitutive reference gene for RT-qPCR across various insect species under diverse physiological conditions [36]. For instance, *Actin* has been identified as one of the most stable reference genes throughout different developmental stages in the leafminer *Liriomyza trifolii* [37]. In the Asian Ladybird *Harmonia axyridis*, *Actin* exhibits minimal variation in Ct values and ranks as one of the three most stable reference genes across sexes [38]. However, mounting lines of evidence demonstrate the significant instability of *Actin* during developmental stages, such as in *H. axyridis* [38], the fall armyworm *S. frugiperda* [14], and the fruit fly *D. suzukii* [23]. Similarly, our study revealed that *Actin* demonstrated the least stability during the larval, pupal, and adult stages (Figure 3 and Figure 4). Furthermore, a poor performance of *Actin* as the reference gene was found in our validation test (Figure 5). Hence, *Actin* might not be the most optimal reference gene for research on the parasitic wasp *T. hagenowii*, particularly concerning different developmental stages. Reference genes within a given insect species are not inherently unique; instead, there often exist multiple candidate genes demonstrating notable stability. Notably, *NADH* displayed remarkable stability across diverse developmental stages in *T. hagenowii*, as indicated by BestKeeper and RefFinder analyses (Figure 4). Similarly, *EF2* also demonstrated relative high stability according to Delta Ct, geNorm, and RefFinder analyses, underscoring its potential as a suitable internal reference (Figure 4). These secondary high-quality candidates have been observed in other insects as well. For instance, *NADH* has been identified as a top-ranking reference gene in the pea aphid *Acyrthosiphon pisum* [39] and in the stink bug *Dichelops melacanthus* [40]. Some commonly used reference genes that are highly stable in developmental stages of other insects, such as *EF-1α* in Lepidoptera, Orthoptera, and Hemiptera insects [14,39,41] and *RP49* in the silkworm *B. mori* [42], were found to exhibit poor performances in *T. hagenowii* (Figure 4). *GAPDH* revealed disappointing expression stability results via RefFinder (Figure 4), which has also been observed during developmental stages in *D. suzukii* [23] and in *H. axyridis* [38]. Overall, in addition to *α-TUB*, *NADH* and *EF2* genes demonstrate stability as reference genes across different developmental stages in *T. hagenowii*.

## Figures and Tables

**Table 1:** Primer sequences, regression coefficient, and amplification efficiency for the RT-qPCR.

Gene Name	Primer Sequence (5′-3′)	Product Length (bp)	Regression Coefficient (R^2^)	Amplification Efficiency (E)
*α-TUB*	F: TACCGTGGTGATGTCGTTCC	206	0.992	95.61%
	R: GCCTCAGCGATAGCAGTTGT			
*EF-1α*	F: CAGATCAGCAACGGCTACAC	198	0.996	90.60%
	R: CTCCTGGAAGGACTCTACGC			
*Actin*	F: GCACCCAGTCCTCCTCACAG	249	0.994	98.10%
	R: CGACCAGCCAAGTCCAAACG			
*RP49*	F: GGAATAGATGCGATAGACAAGCC	223	0.999	96.94%
	R: ATCCACAAATAGTATGGGCTTCA			
*GAPDH*	F: GAGGGTGGTGCCAAGAAAGT	93	0.991	98.15%
	R: TGGCTTGGGTCGTAGGCATCA			
*NADH*	F: GAAGGAGAATGGGCAGTGAA	104	0.995	97.58%
	R: CGATGAGAATACGCCACCAG			
*EF2*	F: GTCGCTGTTGAGCCCAAGAACC	97	0.995	98.79%
	R: CGATGATACATTGGACCATAGGA			

## Data Availability

The data presented in this study are available on request from the corresponding author.

## Supplementary Materials

The following supporting information can be downloaded at: https://www.mdpi.com/article/10.3390/insects15090668/s1; File S1: The raw data of Ct values of the candidate genes evaluated in larvae, pupae, and adults of *T. hagenowii*.
